# Recovery of Flexible Polyurethane Foam Waste for Efficient Reuse in Industrial Formulations

**DOI:** 10.3390/polym12071533

**Published:** 2020-07-10

**Authors:** Gabriel Kiss, Gerlinde Rusu, Francisc Peter, Ionuț Tănase, Geza Bandur

**Affiliations:** 1 University Politehnica Timișoara, Faculty of Industrial Chemistry and Environmental Engineering, C. Telbisz 6, 300001 Timișoara, Romania; gabriel.kiss@momentive.com (G.K.); francisc.peter@upt.ro (F.P.); ionut.tanase@student.upt.ro (I.T.); geza.bandur@upt.ro (G.B.); 2 Momentive Performances Materials, Carl-Duisberg-Strasse 101, 51373 Leverkusen, Germany

**Keywords:** polyurethane foam, recycling, glycolysis, foam properties

## Abstract

Ester polyurethane (PU) foam waste was reacted at atmospheric pressure in an autoclave and using microwaves with diethylene glycol (DEG) at different PU/DEG ratios in the presence of diethanolamine as a catalyst to find the glycolysis conditions that allow for the improved recovery of the PU foam waste and enable the recycling of the whole glycolysis product in foam formulations suitable for industrial application. The recycled polyol was characterized by dynamic viscosity, hydroxyl number, water content, and density, while thermal stability was assessed using thermogravimetric analysis. In the PU foam formulation, 1% and 5% of the glycolyzed material was reused. The relationship between the reuse level of the recycled polyol and the physical properties of the foam was thoroughly investigated. It was observed that both hardness and air flow decreased with increasing recycled polyol content, particularly for the polyester type foam, while tensile strength and compression strength increased. Depending on the amount of recycled polyol and catalyst used, polyether-based foams could be obtained with a low air permeability, needed in special applications as sealed foams, or with higher air permeability desirable for comfort PU foams. The results open the way for further optimization studies of industrial polyurethane foam formulations using a glycolysis process without any separation stage.

## 1. Introduction

Since the polyaddition reaction between an isocyanate and a diol was discovered by Otto Bayer and the invention of polyurethane foams in early 1940′s, the applications of flexible polyurethane foams have been exponentially increasing. The continuous demand of increasing the comfort and improving the lifestyle of the end consumer has always been the driving force of this rapid expansion. Many methods of producing polyurethane foam have been designed, developed, and implemented across the globe [1,2]. End applications such as in bedding, mattresses, upholstery, and the automotive industry take advantage of this material and its unique properties.

In Europe, the polyurethane slabstock (PU) foam yield reached a record high of about more than 1.2 million tons in 2018 to fulfil consumer needs [3]. The global demand of polyurethane products is expected to rise to 22 million tons in 2020 [4], making it the sixth most used polymer in the world [5]. The PU foam market is expected to raise up to USD 74.24 billion by 2021, with an annual growth rate of 8.4% [6]. The synthesis process, along with the conversion process in complex geometries of the PU foam, leads to foam waste that, in extreme situations, reaches up to 20% trim foam. A significant part of the foam waste is reutilized as rebounded foam—filled material for various articles such as pillows, sofas, and others. These methods are well established in the industry, as they allow for a good part of the trim foam to be converted into finished products. However, there is a significant amount of polyurethane foam waste that is unused, ultimately becoming as waste in land-fills.

PU foam waste can be recycled using physical, thermochemical, and chemical methods. The physical recycling methods include regrinding, rebinding, adhesive pressing, injection molding, and compress molding, while the most common chemical methods are hydrolysis, aminolysis, and glycolysis [7]. Glycolysis processes have been reported for a great variety of polyurethane products, including elastomers, coatings, rigid foams, flexible foams, reaction injection molding, reinforced reaction injection molding polyurethanes [5], and even high resilience flexible PU foams [8]. Datta reported the utilization of different low molecular weight glycols and their influence on the structure of the obtained products, carrying out the glycolysis at temperatures between 190 and 250 °C. [9]. Another noteworthy investigation of the group of Professor Datta from Gdansk was focused on the use of crude glycerol, a by-product of biodiesel manufacturing, as a glycolysis agent in the chemical recycling of PU waste. The glycolysis was accomplished in a stainless steel reactor with a mechanical stirrer and a reflux condenser at temperatures between 150 and 220 °C [10]. Borda et al. performed the glycolysis of flexible polyurethane foams and elastomers with ethylene glycol, 1,2-propylene glycol, triethylene glycol, polyethylene glycol, and diethanolamine at 180 °C, and they also proposed a reaction mechanism [11]. Shin et al. obtained a recycled polyol through the glycolysis of waste rigid polyurethane foams scraps, but this recycled product needed further chemical modification by addition polymerization with propylene oxide to deactivate the amine adducts derived from isocyanates [12]. The glycolysis process of rigid polyurethane foams was also carried out with basic catalysts using microwaves as energy sources, and the best results obtained were with potassium hydroxide and sodium hydroxide [13]. Molero et al. used, for the first time, stannous octoate as a catalyst for the glycolysis of flexible polyurethane waste, reporting a higher decomposition rate and purity of polyol than with other catalysts [14]. Recent developments in the field of the chemical recycling of PU waste include the investigation of novel decomposing agents from renewable sources and the utilization of ionic liquids [15].

Though several chemical methods to recover polyurethane foam waste have been described in the literature and numerous patents [16,17,18,19,20,21,22,23,24,25,26], glycolysis processes have not been yet reported as operational at the industrial scale due to economic and/or quality reasons [5]. The high costs associated with several technological drawbacks have hindered this sustainable industrial application. The glycolysis process is usually followed by several separation steps before recycling the polyol, resulting in the need for supplementary equipment and thus increasing cost. Considering the large amount of polyurethane foam waste available for recovery, there is a clear demand to identify new methods or to improve the efficiency of the existing methods.

The aim of this work was to identify improved and economically efficient ways to recycle flexible polyurethane foams by evaluating different glycolysis methods and the reintegration of the recycled material in industrial PU foam formulations. Various glycolysis techniques of polyurethane foam waste were studied, such as an atmospheric pressure method, an autoclave method, and a high frequency method, by assessing the highest possible yield of recycled material. The glycolysis product was used in the same formulations as in the industrial production, targeting the replacement of the main raw material—polyether (or polyester) polyols. The study should allow for a better understanding of the relationship between recovered polyol usage and foam properties. The ultimate goal was to find the optimal conditions from the perspective of raw material recovery, thus introducing the best way to integrate the PU foam waste into the life cycle of industrial scale polyurethane foam production. This could be an important contribution towards the global efforts to reduce carbon footprints, minimize plastic waste material, and eventually contribute to circular economies’ efforts for this specific material.

## 2. Materials and Methods

### 2.1. Glycolysis of the Polyurethane Foam Waste

Flexible polyurethane foam waste, which is based on polyester polyols (Momentive Performance Materials, Leverkusen, Germany), was used as the raw material for the glycolysis process, with diethylene glycol (DEG) as glycolysis agent and diethanolamine (DEOA) as a catalyst. DEG and DEOA were purchased from Sigma Aldrich (Steinheim, Germany).

Three glycolysis methods were investigated.

#### 2.1.1. Glycolysis at Atmospheric Pressure

DEG and the DEOA catalyst were introduced into a 4-neck flask equipped with mechanical stirrer, a distillation column, and a nitrogen blanket. The heating oil bath was equipped with a temperature control unit and a magnetic stirrer. The mixture was gently stirred at a speed of 75 rpm, while the temperature of the bath was gradually increased to 180 °C. The polyurethane foam waste was cut in small pieces not exceeding 0.5 cm in size. Once the temperature reached 160 °C, the first portion of one tenth of polyurethane foam waste was added into the DEG/DEOA mixture. The rest of the foam was added at a rate of one tenth of the foam waste every ten minutes, and then the residual mixture was stirred at 180 °C for another 20 min.

#### 2.1.2. Glycolysis in Autoclave

A stainless-steel autoclave with a 1000 mL total volume (manufactured in University Politehnica Timișoara, Romania), equipped with an adequate sealing system, was employed for the glycolysis at high pressure. In this case, the total amount of PU foam waste was added into the autoclave from the beginning of the experiment, followed by the addition of DEG and DEOA. The autoclave was placed into an oven (Ecocell 111, MMM Medcenter Einrichtungen GmbH, Planegg, Germany) at 180 °C for about two hours.

#### 2.1.3. Glycolysis Using Microwave Equipment

A method similar to the autoclave protocol was employed, with the main difference being that a microwave digestion system (Speedwave MWS-2, Berghof, Germany) equipped with high-pressure teflon reactors of 60 mL capacity, without stirring, was used instead of a standard oven [14]. The glycolysis was carried out at 190 °C, for 10 min.

#### 2.1.4. Characterization Methods of the Glycolysis Products

The water content (%) (using ASTM D4672-18), viscosity (cSt) (using ASTM D4878-15), hydroxyl number (mg KOH/g) (using ASTM D4274-16), and density (g/cm^3^) (using ASTM D4669-18) of the glycolysis products were evaluated according to well-established methods used in most polyurethane factories.

### 2.2. Synthesis of Polyurethane Foams Using Recycled Polyol

The glycolyzed products were used as raw materials in the flexible polyurethane foam production. A variable part of the virgin polyol was replaced by the glycolysis product in both ester and ether polyurethane foam formulations.

#### 2.2.1. Flexible Polyurethane Ester Foam

The polyester foam formulation presented in Table 1 was selected to investigate the influence of the recycled polyol in different reaction conditions. The equipment used for foam preparation was a standard bench mixing station (manufactured by Pendraulik Maschinen und Apparate GmbH, Springe, Germany) with a variable rotation speed that was equipped with a standard impeller and a rate of rise system (Format Messtechnik, Karlsruhe, Germany).

Desmophen 2200B (virgin polyol) from Covestro (Leverkusen, Germany) and toluene diisocyanate (TDI 80/20) (available under the commercial name Lupranate T-80 from BASF (Ludwigshafen, Germany)) were used as main raw materials. The amine catalysts, Niax catalyst C-131NPF and Niax catalyst DMP, along with the silicone surfactant Niax Silicone L-537XF, were supplied by Momentive Performance Materials (Leverkusen, Germany). All these raw materials, except for the recycled polyol, are commercially available.

#### 2.2.2. Flexible Polyurethane Ether Foam

The selected polyether foam formulation is presented in Table 2, and the influence of the recycled polyol on the foam properties was assessed in different foam conditions. The equipment used for the foam preparation was the same as described for the polyester-based foam. Voranol 3322 (virgin polyol) from Dow Chemicals (Midland, MI, USA) and toluene diisocyanate were used as main raw materials. The catalysts, Niax catalyst B-18, Niax catalyst A-1, and Niax Stannous octoate, along with the surfactant Niax Silicone L-595, were supplied by Momentive Performance Materials (Leverkusen, Germany).

#### 2.2.3. Physical Properties of the Foams

The same methods were used for the determination of physical characteristics of the obtained polyether- and polyester-based foams. Foam density was measured on 10 × 10 × 5 cm^3^ foam samples according to DIN 53420. Compression force deflection (CFD) at 40%, expressed in kilopascals (kPa), was measured on 10 × 10 × 5 cm^3^ foam samples, according to the ISO3386/1 test method. Foam porosity or airflow, expressed in liters per minute (L/min), was measured on 5 × 5 × 2.5 cm^3^ foam samples, according to the ISO7231 test method. The cell structure was characterized by visual observation [27].

## 3. Results and Discussion

### 3.1. Comparative Evaluation of the Glycolysis Methods

Three different glycolysis procedures were investigated to select the method that led to the best result of the recovered polyurethane material—glycolysis at atmospheric pressure—in the autoclave and using microwave heating. The main aim was to investigate the influence of the glycolysis methods on the decomposition ratio of the polyurethane foam waste and to characterize the glycolyzed products based on main specification characteristics such as viscosity, hydroxyl number, and water content. Ultimately, the objective was to use the glycolyzed material in the polyurethane foam formulations and compare the advantages and shortcomings of each method. It is important to mention that all the three glycolysis methods used the same type of foam waste, as well as the same batches of DEG and DEOA, to minimize potential variations. It was also important to evaluate which method enabled the best balance between the yield of the recovered polyol and the possible industrial implementation, specifically looking to the foam property improvements when a small quantity of recycled polyurethane was used.

Foam waste generated from polyester-based polyurethane foam was used for the glycolysis process. Such foams are widely used in the automotive industry thanks to their strong flame bonding property on various substrates [27,28] and outstanding tensile and elongation properties. The ester slabstock foams are used for textile applications (flame lamination), packaging, filters, etc. This type of foam has inferior comfort characteristics and is hydrolytically less stable than the polyether-based polyurethane foam, so it is not used for seating or bedding applications [28]. Regarding concerns of the glycolysis agent, the reason for choosing DEG was its good compatibility with the aforementioned foam waste, increasing the chances of high recovered product yields. The boiling point of DEG is 245 °C, enabling us to conduct glycolysis at high temperatures without a loss of raw material. The purity of DEG is also important to avoid eventual side reactions.

The stepwise addition of the foam waste was necessary in the glycolysis procedure at atmospheric pressure because foam waste absorbs the raw materials used for glycolysis, resulting in a swelling of the foam that could impede mixing. To avoid that, one tenth of the foam waste was added every ten minutes. In this way, the polyurethane foam degradation proceeded acceptably. However, mixing difficulties were observed after every addition stage, and in the last foam waste addition sequences, the difficulties were even greater. On the contrary, the glycolysis using the autoclave did not need stepwise addition and was not hindered by mixing difficulties, even allowing for the utilization of higher PU waste/DEG ratios, as is discussed in Section 3.3. The third method, glycolysis using a microwave equipment, enabled a much shorter time to complete the decomposition, but a potential industrial application would involve an increased equipment cost and a high energy consumption.

Table 3 shows the results obtained by the three different glycolysis procedures. All three methods allowed for the decomposition of the PU foam waste, resulting in a homogeneous brown liquid (Figure S1, Supplementary Material) with typical characteristics. Three experiments (designed as EXP. 1, EXP. 2, and EXP. 3) were run using the same formulation, with 25% (weight) PU waste and 75% DEG. All methods led to stable recycled material. As previously discussed, the sequential addition of polyurethane foam waste slowed down the whole process, leading to a long glycolysis time. The glycolysis test using the microwave (EXP. 3) was a much faster process that led to a recycled polyol with similar characteristics, but it was a more complex procedure that generated higher costs.

### 3.2. Flexible Polyurethane Foam Production Using Glycolyzed Products as Raw Materials

The glycolyzed products were used as raw materials for the production of both polyester-based and polyether-based flexible polyurethane foams. In all experiments, 1% of virgin polyol was replaced by the glycolysis products, and the impact of this recycled product on the physical properties of the polyurethane foams was studied.

#### 3.2.1. Production of Polyester-Based Flexible Polyurethane Foams

The polyester foam formulations presented in Table 1 are the original developments accomplished during this work. They are laboratory-scale formulations sufficiently close to those used in current industrial practices that deliver the best foam performances for polyester-based slabstock foam production, such as fine cell structure, stable foam, and good foam porosity. At the same time, the developed formulation was sensitive enough to allow us to identify possible shifts of performances (improvement or degradation of properties), compared with a foam made with virgin polyol only.

Table 4 shows the results of using the ester foam formulation from Table 1. The reference formulation was made using 100 parts virgin polyol. In the experiments, 1% virgin polyol was replaced by recycled polyol obtained from the glycolysis experiments EXP. 1–3. Various properties were analyzed in a back-to-back comparison relative to the reference foam. The rise time was similar in all different foams, indicating no deviation when a recycled polyol was used. A second important parameter was the so-called “foam settling.” This is a critical parameter that enables the detection of foam instability or over stabilization. The results in Table 4 indicate a minimal stability shift, regardless of the recycled polyol used.

Furthermore, the foam properties were measured after 24 h, thus allowing for a full curing of the polyurethane foams. The foam density of the reference foam was 25.1 kg/m^3^. The addition of the recycled polyol determined a drop of the foam density with 0.6 kg/m^3^ in average kg/m^3^. It must be pointed out that a lower density foam using the same total amount of raw materials would turn into a significant benefit, as, in most cases, the foam is sold per volume or per unit; thus, a lower density foam would generate an economical advantage.

The hardness of the reference foam was 4.8 kPa, representing a typical value for such a foam. The addition of recycled polyol resulted in a somewhat softer foam when using all three products from the glycolysis experiments. The loss of foam hardness was believed to be linked to finer cell structure when using the recycled polyols. A finer cell structure is preferred in textile application.

Compression sets (expressed in percent) represent the ratio between the initial thickness and the foam thickness after compression treatment. A lower compression set of a polyurethane foam indicates a better foam recovery. As shown in Table 4, the addition of recycled polyol slightly increased the compression sets.

Foam airflow is the parameter that indicates the close cell versus open cell content of the polymer. Low values usually indicate a high closed cell content, while higher values indicate the opposite. Flexible polyurethane foams are characterized by a lower level of close cell content, allowing for a good breathability. The results in Table 4 point out that the recycled polyols slightly decreased the airflow compared to the reference.

Tensile strength is another important characteristic for the quality of polyurethane foam, representing the force needed to break polyurethane foam specimen. Therefore, a higher number indicates an improved performance for this specific characteristic. In this case, all foams made with recycled polyol indicated an improvement of this property.

A cell structure assessment is a subjective ranking of the size and uniformity of foam cells. Lower numbers indicate finer and smaller cell size, while higher numbers suggest a coarser and irregular cell size. The addition of recycled polyol improved the cell structure towards a finer cell size, as previously mentioned. A smaller cell size is preferred in textile and flame lamination foams [27].

#### 3.2.2. Production of Polyether-Based Flexible Polyurethane Foams

The recycled polyol was also used in polyether-based foam applications as a partial replacement of virgin polyol. The polyether foam formulations shown in Table 2 were also developed in-house. The raw materials differed from the ester foam; only the isocyanate and the recycled polyols were the same. A 4.5 parts water content was selected for the polyether foam formulation, because it would lead to a density of 25 kg/m^3^ or slightly lower, which is considered the average density for polyether foam formulations.

Table 5 presents the properties of the foams obtained using the ether foam formulation described in Table 2. As in the case of the polyester-based PU foams, 1% recycled polyol from EXP. 1–3 was added, replacing the virgin polyol. The same properties as the ester type formulation were assessed after 24 h from the foam production using similar testing protocols.

The density of the reference foam was 23.2 kg/m^3^. The addition of recycled polyol determined a decrease of the foam density with 0.3 kg/m^3^ in average.

The reference foam exhibited a 3.34 kPa hardness value, which typical for this specific formulation. The addition of recycled polyol (EXP. 1–3) enabled the manufacturing of harder foam without any effect on the cell structure. Typically, an increase of foam hardness can be achieved with the use of specially-grafted polyols, so called styrene-acrylonitrile co-polymer polyols, which, by their nature and production process, would bring additional cost to the foam [29]. Compared to the other recycled polyols, the highest hardness increase was observed when the glycolysis was performed in the autoclave.

Table 5 also shows that the addition of recycled polyol seemed to not have a real influence on the compression sets but led to decrease of the foam airflow. In this case, the airflow shift was higher than in the case of the polyester foams with recycled polyol. Identical to the ester foams, adding recycled polyol resulted in an improvement of the tensile strength. Unlike the ester foams, where the addition of recycled polyol shifted the cell structure towards being finer, adding any of the recycled polyols had no impact on the cell structure in the case of the ether foams.

### 3.3. Detailed Evaluation of the Glycolysis of the Polyurethane Foam Waste Using the Autoclave Method

Though the results presented in Section 3.2.1 and Section 3.2.2 are close, they ultimately show the glycolysis in the autoclave as the best among the three studied methods for the forthcoming studies. Glycolysis at atmospheric pressure has a limitation of yield (it does not allow for a further increase of the foam waste content), while the microwave technology is expected to be a high energy consumer. The foam performance improvements refer specifically to (i) density improvement, as a lower density compared to the reference foam means an economical advantage, and (ii) a higher tensile strength than for the reference foam, meaning a more resistant foam. Therefore, the autoclave method was identified as the best performing in terms of glycolysis procedure and improved foam properties for both polyester-based and polyether-based polyurethane foam formulations. The next step was to investigate the possibility to increase the amount of foam waste subjected to the glycolysis process using this method. The influence of foam waste quantity allowed us to define the best process conditions, identifying the critical processing edges and targeting the highest possible recycled foam amount.

#### 3.3.1. Influence of the Polyurethane Foam Waste Amount

Various PU foam waste: DEG ratios between 1:3 and 1:1 were used in the autoclave glycolysis procedure. In all three experiments a liquid glycolysis product was obtained, and the characteristics are presented in Table 6.

As expected, an increase of polyurethane foam waste material led to a higher viscosity. At a 1:3 ratio, the viscosity was rather low. With the increase of the foam waste content, the viscosity increase was significant, while the hydroxyl number dropped, not necessarily respecting the linearity of the viscosity characteristics. It is important to note that a slight water content increase was observed as a linear response to the increase of foam waste in the glycolysis mixture (Table 6). The polyurethane foam waste could be considered a carrier of water or moisture. It must be also pointed out that the water content was part of the polyurethane foam formulation; therefore, a recalculation of the added water will be necessary when using such recycled polyol. Alternatively, a drying procedure could be applied prior to glycolysis, which would come with additional energy consumption for the overall procedure.

Using the autoclave method, the degradation of 1 part polyurethane waste was possible with only 1 part DEG, which was a much higher yield compared to the atmospheric pressure method. Since all glycolysis products showed appropriate properties for utilization in polyurethane foam formulations, they were investigated for the manufacturing of both polyether-based and polyester-based polyurethanes, targeting the possible increase of the recycled material.

#### 3.3.2. Thermogravimetric Analysis of the Autoclave Glycolysis Products

**Figure 1:**
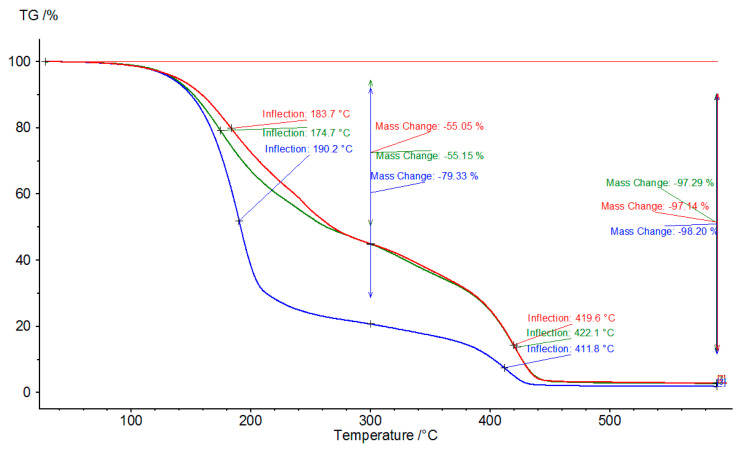
TGA curves of the glycolysis products obtained by the autoclave method at different PU waste/DEG weight ratios: 1:3 (EXP. 2; blue line), 1:2 (EXP. 4; red line), and 1:1 (EXP. 5; green line).

In order to study the thermal stability of the samples, TGAs were performed (Figure 1). The weight loss until 100 °C was under 1% and was usually associated with the water content. These data are consistent with the water content of the samples from Table 6. The thermal decomposition of the samples occurred in two steps. The first step between 100 and 240 °C could be associated with the loss of small molecule compounds. EXP. 2 had the highest mass loss (79.32%) at 300 °C, which was consistent with the highest hydroxyl number (which denotes smaller molecules with lower boiling points) and the lowest viscosity. The samples from EXP. 4 and 5 had similar weight losses at 300 °C and comparable hydroxyl numbers and viscosities. The second degradation step was between 350 and 460 °C and was associated with the degradation of the glycolysis products. The final residue at 500 °C was around 2%.

### 3.4. Influence of the Polyurethane Foam Waste Quantity and its Use Level on the Properties of the Ester Foam

The aim of this study was to determine the behavior of recycled polyol in relationship with its use level and polyurethane foam waste content. Table 7 contains the properties of the ester foams obtained with recycled polyols replacing 1% and 5% virgin polyol in the formulation. Tendentially, at 1 part of recycled polyol, a decrease of density with a softening effect, an improved tensile strength, and a smaller cell structure were observed for all recycled polyol compositions. The increase of recycled polyol to 5 pbw somehow triggered different behaviors in the foam properties. The reactivity profile changed, leading to slightly faster rise time, while the foam settling increased from an average of 1.2% to an average of 7–9%. The combined effect between decreased rise time and increased foam settling indicated a destabilizing effect. While at 1 part recycled polyols, improvements of most characteristics were achieved, a too-high level seemed to affect the stability of the ester foam formulation. Compression sets were also affected by the increased level of recycled polyol, while tensile strength showed a strong improvement. The foam cell structure became finer with the increase of the recycled polyol content.

Since each of the recycled polyols performed similarly, EXP. 5, containing higher foam waste content, was preferred due to its better overall performance.

### 3.5. Influence of the Polyurethane Foam Waste Quantity and its Use Level on the Properties of the Ether Foam

Table 8 presents the properties of the ether foams obtained with recycled polyols at 1 and 5 parts. At a low use level of recycled polyol, improvements such as a reduced foam density, an increased hardness, and an improved tensile strength were observed. The increase of recycled polyol to 5 parts, however, triggered different outcome than for the ester foam evaluation. Unlike in the ester foam, the addition of recycled polyol did not affect the reactivity profile or foam settling. Compression sets shifted with the increase of the added recycled polyol amount, in the same way as in the previous example. The tensile strength also showed a strong improvement.

One of the most impacted characteristics was the foam airflow. Polyether polyurethane foams are characterized as high foam-openness materials if the intention is to be used in bedding industry. The addition of 5 parts of recycled polyol dropped the airflow to below 10 L/min. Such a polyurethane foam will be suitable for special applications, such as sealed foams, that are characterized by a very low air permeability. This is an outstanding feature that only normally can be achieved by using special raw materials. Besides very low airflow, another requirement for these applications is to be free of foam shrinkage. The combined effects of a very low airflow with a lack of foam shrinkage were observed for the foams made with EXP. 4 and 5 at a higher recycled PU waste content. The foam made with EXP. 2 also enabled a low airflow, but a strong foam shrinkage was observed (Figure S2, Supplementary Material). Considering the overall foam performances, specifically the foam shrinkage, EXP. 2 will likely be excluded for such an application, whereas EXP. 4 and 5 outperformed and could be considered candidates for sealed foam applications. It can be concluded that for such applications, a recycled polyol with higher content of polyurethane foam waste is recommended. In our studies, recycled polyols with both 33.3% (EXP. 4) and 50% (EXP. 5) polyurethane foam waste content performed well.

### 3.6. Optimization of the Ether Foam Formulation with Recycled Polyol by Selection of the Catalyst

The results presented in the previous sections demonstrated that a small amount of recycled polyol can selectively improve foam properties, both in ether foam and ester foam applications. At the same time, an increase of recycled polyol, specifically in ether foam formulation, could be suitable for low air permeability foam types. However, if the targeted application is comfort foams, there is big challenge to increase the recycled polyol content without affecting the foam properties, especially foam airflow. A potential technical solution to address this shortcoming could be foam formulation optimization through selecting an appropriate catalyst type and amount. Table 9 presents the results of this study. The reference foam based on 100 parts virgin polyol resulted in standard foam properties. The airflow value was 109 L/min, a typical value characterizing open cell polyurethane foam. The addition of 5 parts of recycled polyol in EXP. 5 to replace the virgin polyol hugely dropped the airflow to 5 L/min. An optimization of the formulation was carried out by the improved selection of the amine catalyst along with the proper adjustment of the stannous octoate level (EXP. 6). In order to favor the blowing reaction and consecutively enable an increased foam airflow. Niax Catalyst A-1, known as strong blowing catalyst, was tested instead of Niax Catalyst B-18. The optimum catalyst level was identified to be 50% less in amount. Moreover, reduction of the Niax Stannous octoate amount from 0.18% to 0.12% was also identified as a solution for the optimum catalyst package. These foam formulation adjustments allowed for the recovery of airflow characteristics similar to the reference foam (Table 9 and Figure S2, Supplementary Material). In conclusion, the right selections of raw materials and additives are the key issues to realize an increased use level of recycled polyol in polyurethane foam formulations. Considering this approach, a further increase of recycled polyol could possibly be attained, but a more in-depth evaluation was outside the scope of this study.

## 4. Conclusions

This work led to a better understanding and detailed comparison of various glycolysis procedures, ultimately identifying the most optimum process. The recycled polyols can be used as raw materials to replace virgin polyols in polyurethane foam formulations in both ester and ether applications. The autoclave method proved to be the best glycolysis technique when diethanolamine was used as a catalyst, allowing for an increased use level of foam waste and improving overall properties. The utilization of recycled polyol at a low level resulted in an improved foam density for both ester and ether foams. The effect on foam hardness was different, in the ester foam the recycled polyol enabled a softening effect, while in the ether foam led to a somewhat higher hardness. In both ester and ether foams, a tensile strength improvement was observed. Generally, the increased use level of recycled polyol affected the foam compression sets. In the case of ether polyurethane foam, a lower air permeability foam could be achieved using a higher amount of recycled polyol. These foams could be used in special applications where low air permeability is necessary. Meanwhile, process optimization allowed for the increase of the recycled polyol amount and a higher airflow. The right selection of the formulation recipe with appropriate adjustments allowed for an increase of up to of 5% the recycled polyol amount in the foam composition. Though this amount was lower than reported in the literature when using purified recovered polyols, it has the important advantage of eliminating any separation steps and recycling the whole glycolysis product. Based on these findings, the next step will be to target an even higher quantity of recycled polyols with the specified composition in polyurethane formulations and to identify the best foaming conditions.

## Figures and Tables

**Table 1:** Formulation of the polyester foam used in this study.

Foam Formulation	pbw *
Desmophen 2200B	100 **
Recycled polyol	vary
Water	4.90
Niax catalyst C-131 NPF	1.00
Niax catalyst DMP	0.15
Niax silicone L-537XF	1.50
Toluene diisocyanate (TDI 80/20)	51.00

* pbw = parts by weight (used in industrial polyurethane (PU) formulations); ** Desmophen 2200B quantity was recalculated for every synthesis, in function of the added recycled polyol.

**Table 2:** Formulation of the polyether foam used in this study.

Foam Formulation	pbw *
Voranol 3322	100 **
Recycled Polyol	vary
Water	4.50
Niax catalyst B-18 (Niax catalyst A-1 ***)	0.15
Niax Silicone L-595	1.00
Niax Stannous octoate	0.18
TDI 80/20	55.00

* pbw = parts by weight (used in industrial PU formulations); ** Voranol 3322 quantity was recalculated for every synthesis in function of the added recycled polyol; *** used only in Section 3.6

**Table 3:** Formulation and comparative results of the three glycolysis types. PU: polyurethane; DEG: diethylene glycol; DEOA: diethanolamine.

Glycolysis Method	Atmospheric Pressure	Autoclave	Microwave
**Experiment Number**	EXP. 1	EXP. 2	EXP. 3
PU Foam waste (%)	25.0	25.0	25.0
DEG (%)	75.0	75.0	75.0
DEOA (%)	0.5	0.5	0.5
Temperature (°C)	180–190	180.0	190.0
Reaction time (min)	120.0	120.0	10.0
Water content (%)	0.202	0.372	0.457
Viscosity (cSt)	179.0	172.0	167.0
Hydroxyl number (mg KOH/g)	823.0	869.0	784.0
Density (g/cm^3^)	1.12	1.14	1.17

**Table 4:** Properties of the foams obtained using the ester formulation presented in Table 1, replacing 1 pbw virgin polyol (Desmophen 2200B) with recycled polyol. CFD: compression force deflection.

Polyol Used for the Ester Foam.	Reference	EXP. 1	EXP. 2	EXP. 3
**Desmophen 2200B (virgin polyol) (%)**	100.0	99.0	99.0	99.0
Recycled polyol (%)	0.0	1.0	1.0	1.0
**Foam Physical Properties**				
Rise time (s)	119.0	117.0	118.0	117.0
Foam settling (%)	1.20	2.10	2.00	1.50
Density (kg/m^3^)	25.10	24.46	24.48	24.25
Hardness CFD-40% (kPa)	4.80	4.42	4.46	4.24
Compression set 22 h 75% 70 °C (%)	8.00	9.85	9.70	10.05
Airflow (L/min)	33.0	21.0	25.0	25.0
Tensile strength (kPa)	107.0	144.0	124.0	132.0
Cell structure (fine 1.... coarse 8)	5.0	4.0	4.0	4.0

**Table 5:** Properties of the foams obtained using the ether formulation presented in Table 2, replacing 1 pbw virgin polyol (Voranol 3322) with recycled polyol.

Ether Foam	Reference	EXP. 1	EXP. 2	EXP. 3
Voranol 3322 (virgin polyol) (%)	100.0	99.0	99.0	99.0
Recycled polyol (%)	0.0	1.0	1.0	1.0
**Foam Physical Properties**				
Rise time (s)	90.00	88.00	87.00	90.00
Foam settling (%)	0.60	0.70	0.80	0.80
Density (kg/m^3^)	23.20	22.90	22.80	23.20
Hardness CFD-40% (kPa)	3.34	3.40	3.44	3.38
Compression 22 h 75% 70 °C (%)	17.86	14.40	17.70	17.50
Airflow (L/min)	109.0	76.0	75.0	73.0
Tensile strength (kPa)	92.0	99.0	98.0	98.0
Cell structure (fine 1.... coarse 8)	2.0	2.0	2.0	2.0

**Table 6:** Influence of the polyurethane foam waste quantity on the recycled polyol characteristics using autoclave glycolysis.

Glycolysis Method	Autoclave	Autoclave	Autoclave
**Experiment Number**	EXP. 2	EXP. 4	EXP. 5
PU Foam waste (%)	25.0	33.3	50.0
DEG (%)	75.0	66.7	50.0
DEOA (%)	0.5	0.5	0.5
Temperature (°C)	180.0	180.0	180.0
Time (min)	120.0	120.0	120.0
**Characteristics of the Recycled Polyol**			
Water content (%)	0.372	0.673	0.774
Viscosity (cSt)	172.0	1458.0	2502.0
Hydroxyl No. (mg KOH/g)	869.0	620.0	593.0
Density (g/cm^3^)	1.14	1.15	1.15

**Table 7:** Properties of the ester foams obtained using the formulation indicated in Table 1, replacing 1% or 5%. virgin polyol with recycled polyol.

Ester Foam	Ref.	EXP. 2	EXP. 4	EXP. 5	EXP. 2	EXP. 4	EXP. 5
Desmophen 2200B (virgin polyol) (%)	100.0	99.0	99.0	99.0	95.0	95.0	95.0
Recycled polyol (%)	0.0	1.0	1.0	1.0	5.0	5.0	5.0
**Foam Physical Properties**							
Rise time (s)	119.0	118.0	117.0	117.0	113.0	111.0	112.0
Foam settling (%)	1.20	2.00	1.90	1.70	7.90	6.70	9.00
Density (kg/m^3^)	25.10	24.50	24.80	24.30	25.10	25.60	25.50
Hardness CFD-40% (kPa)	4.86	4.46	4.62	4.60	3.40	3.75	3.82
Compression set 22 h 75% 70 °C (%)	7.90	9.70	8.72	8.66	24.78	26.37	21.02
Initial air flow (L/min)	33.0	25.0	27.0	27.0	14.0	11.0	9.0
Tensile strength (kPa)	107.0	124.0	121.0	117.0	156.0	161.0	155.0
Cell structure (fine 1.... coarse 8)	5.0	4.0	4.0	4.0	3.0	3.0	3.0

**Table 8:** Properties of the ether foams obtained using the formulation indicated in Table 2, replacing 1% or 5% virgin polyol with recycled polyol.

Ether Foam	Reference	EXP. 2	EXP. 4	EXP. 5	EXP. 2	EXP. 4	EXP. 5
Voranol 3322 (virgin polyol) (%)	100.0	99.0	99.0	99.0	95.0	95.0	95.0
Recycled polyol (%)	0.0	1.0	1.0	1.0	5.0	5.0	5.0
**Foam Physical Properties**							
Rise time (s)	89.0	87.0	89.0	88.0	89.0	89.0	88.0
Foam settling (%)	0.60	0.80	0.60	0.80	0.50	0.70	0.80
Density (kg/m^3^)	23.13	22.84	22.99	22.96	21.58	21.36	21.60
Hardness CFD-40% (kPa)	3.34	3.44	3.41	3.48	3.22	3.43	3.34
Compression 22h 75% 70 °C (%)	17.86	20.73	20.64	18.70	62.17	54.94	54.61
Initial air flow (L/min)	109.00	75.00	84.00	81.00	0.50	3.00	5.00
Tensile strength (kPa)	92.0	98.0	102.0	111.0	59.0	95.0	94.0
Cell structure (fine 1.... coarse 8)	2.0	2.0	2.0	2.0	3.0	3.0	3.0
Foam shrinkage	no	no	no	no	yes	no	no

**Table 9:** Optimization of the process parameters for the ether foam formulation and properties of the foam obtained with 5 pbw recycled polyol.

Ether Foam	Reference	EXP. 5	EXP. 6
Voranol 3322 (virgin polyol) (%)	100.0	95.0	95.0
Recycled polyol (%)	0.0	5.00	5.00
Niax Catalyst B-18 (%)	0.15	0.15	–
Niax Catalyst A-1 (%)	–	–	0.075
Niax Stannous octoate (%)	0.18	0.18	0.12
**Foam Physical Properties**			
Rise time (s)	89.80	88.40	94.10
Foam Settling (%)	0.60	0.80	1.30
Density (kg/m^3^)	23.13	21.60	22.66
Hardness CFD-40% (kPa)	3.34	3.34	2.74
Compression set 22h 75% 70 °C (%)	17.89	54.60	22.70
Initial air flow (L/min)	109.0	5.0	113.0
Tensile strength (kPa)	33.0	27.0	14.0
Cell structure (fine 1.... coarse 8)	2.0	2.0	2.0

## Supplementary Materials

The following are available online at https://www.mdpi.com/2073-4360/12/7/1533/s1, Figure S1: Glycolysis product obtained from flexible PU foam waste and diethylene glycol at 1:1 (*w/w*) ratio in an autoclave (image at right), compared to the virgin polyol (image at left); Figure S2: Images of the reference polyether-based polyurethane foam (a); polyether-based polyurethane foams obtained with 1% recycled polyol from 25% PU waste (EXP. 2) (b), respectively, from 50% PU waste (EXP. 5) (c); polyether-based polyurethane foams obtained with 5% recycled polyol from 25% PU waste (EXP. 2) (d), respectively, from 50% PU waste (EXP. 5) (e); polyether-based polyurethane foams obtained with 5% recycled polyol from 50% PU waste and optimized formulation (EXP. 6) (f).
