# Supplementary material for: Recovery of Flexible Polyurethane Foam Waste for Efficient Reuse in Industrial Formulations
Source: Polymers (Basel). 2020 Jul 10;12(7):1533. doi: 10.3390/polym12071533 (PMC7407941; doi:10.3390/polym12071533)
Supplement: Supplementary file 1 [file polymers-12-01533-s001.pdf]

Supplementary material

Recovery of flexible polyurethane foam waste for efficient reuse in industrial formulations

Gabriel Kiss [1,2], Gerlinde Rusu [1*], Francisc Péter [1], Ionuț Tănase [1] and Geza Bandur [1]

[1] University Politehnica Timișoara, Faculty of Industrial Chemistry and Environmental Engineering, C. Telbisz 6, 300001 Timișoara, Romania

[2] Momentive Performances Materials, Carl-Duisberg-Strasse 101, 51373 Leverkusen

* Correspondence: Gerlinde.rusu@upt.ro; Tel.: +40-256-404221

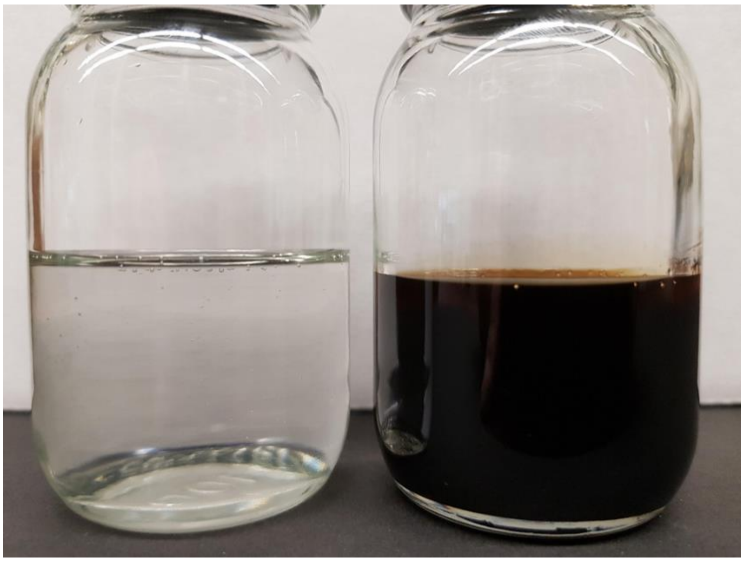

Figure 1S. Glycolysis product obtained from flexible PU foam waste and diethylene glycol at 1:1 (w/w) ratio, in autoclave (image at right), compared to the virgin polyol (image at left)

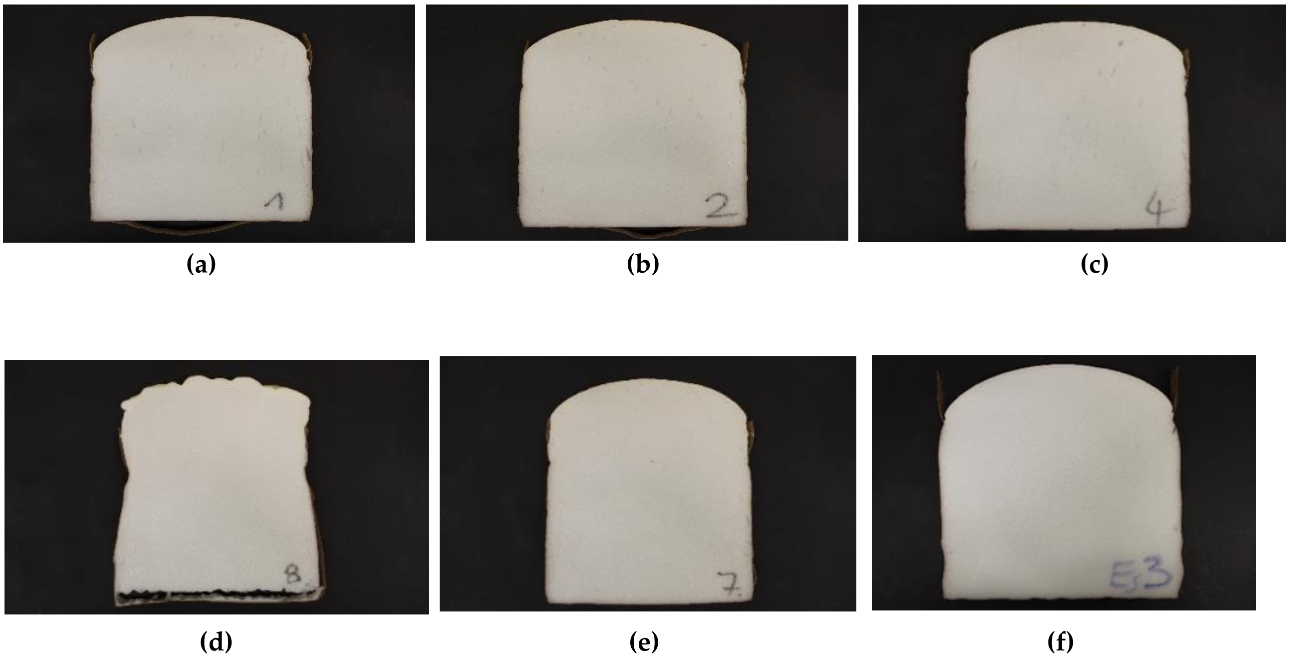

Figure 2S. Images of the reference polyether-based polyurethane foam (a); polyether-based polyurethane foams obtained with 1% recycled polyol from 25% PU waste EXP 2 (b), respectively from 50% PU waste EXP 5 (c); polyether-based polyurethane foams obtained with 5% recycled polyol from 25% PU waste EXP 2 (d), respectively from 50% PU waste EXP 5 (e); polyether-based polyurethane foams obtained with 5% recycled polyol from 50% PU waste and optimized formulation EXP 6 (f)
